# KRAS above and beyond – EGFR in pancreatic cancer

**DOI:** 10.18632/oncotarget.750

**Published:** 2012-11-13

**Authors:** Jens T. Siveke, Howard C. Crawford

**Affiliations:** II. Medizinische Klinik, Klinikum rechts der Isar, Technical University of Munich; Department of Cancer Biology, Mayo Clinic Florida, Jacksonville, FL

Pancreatic ductal adenocarcinoma (PDAC) remains one of the most lethal of all malignancies with a frightening resistance to chemotherapeutic and targeted approaches. Recent evidence defines how EGF receptor is involved in tumor formation, which may lead to novel approaches for PDAC prevention and therapy.

Besides lung cancer and some colon cancers, PDAC is one of the few primarily oncogene-driven solid tumors, with oncogenic *KRAS* mutations found in up to 95% of cases. Along with the central role of KRAS-dependent signaling, receptor tyrosine kinases and especially the EGF receptor (EGFR) and its ligands are strongly upregulated in PDAC and chronic pancreatitis (CP), a risk factor for PDAC. The relevance of this upregulation was hinted at with the advent of a transgenic mouse model overexpressing TGFA, one of the main EGFR ligands. These mice develop extensive fibrosis and display a type of epithelial morphogenesis frequently associated with PDAC and CP, known as acinar-to-ductal metaplasia (ADM). However, these mice progress to PDAC only rarely, unless crossed into a p53 null background [1]. In vitro studies show that treatment of acinar cells with EGFR ligands induces a phenotypic conversion to a duct-like cell, a process later confirmed to be true ADM [2]. The role of ADM as a precursor to PDAC has been confirmed in multiple studies since then using various genetically engineered mouse models (GEMM) (reviewed in [3]) and in human carcinogenesis [4]. Thus, supraphysiological EGFR activation reprograms the supposedly terminally differentiated acinar cell to a preneoplastic ductal lesion.

The importance of the endogenous EGFR in PDAC tumorigenesis was largely dismissed since one of its major downstream targets is KRAS, which, when mutated, should no longer need stimulation by upstream components. Indeed, GEMMs confirm that oncogenic KRAS is sufficient to induce ADM, PanIN and eventually invasive and metastatic PDAC. Interestingly however, when oncogenic KRAS and TGFA overexpression are combined, ADM, PanIN and PDAC formation is greatly accelerated [5], indicative of either an incomplete overlap between KRAS and EGFR signaling or with EGFR enhancing the efficiency of transformation, perhaps by inducing transformation-sensitive ADM.

To directly define the impact of EGFR signaling in a setting of oncogenic KRAS signaling, we generated mice with conditional deletion of *Egfr* concomitant with *Kras^G12D^* expression [6]. Surprisingly, these mice showed virtually no neoplastic lesions, consistent with KRAS^G12D^ recruiting EGFR for its ADM-inducing activity. Interestingly, deletion of the primary EGFR ligand sheddase, *Adam17*, showed a similar protection, indicating that the EGFR ligands responsible for this critical EGFR activation originate from the parenchyma rather than the stroma, even in the context of pancreatitis-induced tumorigenesis. This latter result was particularly surprising, since the mechanism of pancreatitis-induced PDAC is naturally assumed to be the result of interactions with inflammatory cells rather than by cell autonomous mechanisms, perhaps indicating some important limitations in our current models of pancreatitis. Remarkably, Navas and colleagues showed using a similar approach that, unlike the pancreas, *Kras^G12V^*-driven tumor development in lung and colon GEMMs did not rely on EGFR signaling, providing further strong evidence for a unique EGFR-mediated process in the pancreas [7].

**Figure:**
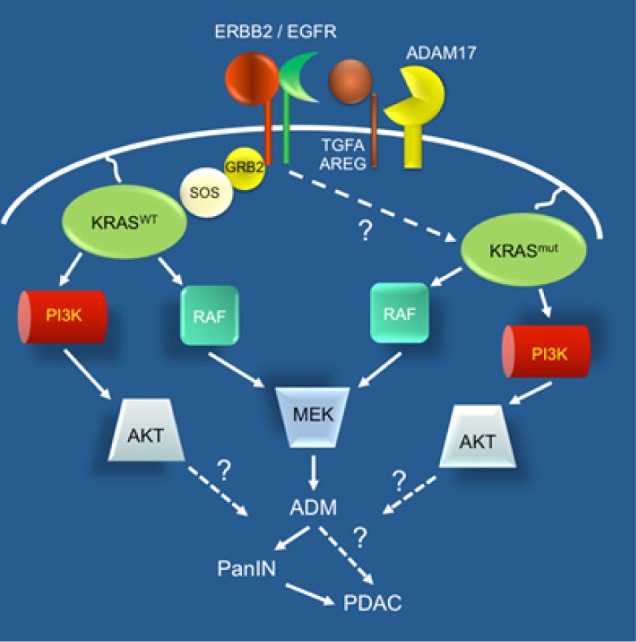


What is the critical function of EGFR in pancreatic tumorigenesis? *Egfr* knockouts consistently had lower levels of MEK/ERK signaling and pharmacological inhibition of MEK effectively ablated tumor initiation *in vivo* and ADM *in vitro*. But why would MEK need EGFR for its activation in a KRAS mutant background? Recent studies of Logsdon and Ji have shown that pancreatic tumorigenesis is strongly dependent on a minimal threshold of KRAS activity, which is not achieved simply by a single mutant *Kras* allele [8]. Indeed, consistent with their observations, we found that ADAM17 activation of EGFR was required for robust KRAS activity in acinar cells. Still, many open questions remain: Are there critical pathways that KRAS cannot directly affect that are compensated for by EGFR activation? How does mutant KRAS upregulate EGFR? Is inflammation the source of EGFR ligand in a less artificial model of pancreatitis-induced cancer? Whatever the answers, the requirement of EGFR activation for the very initial steps in pancreatic carcinogenesis opens the door for preventive approaches targeting EGFR and MEK/ERK signaling, e.g. in patients at high risk of developing PDAC.

What role does EGFR play once PDAC has developed? This question is far more difficult to address experimentally using GEMMs. Clinically, only a subgroup of PDAC patients, those developing a rash upon erlotinib treatment, benefits from an EGFR-targeted therapy [9]. However, predictive biomarkers that determine benefit from erlotinib treatment have not yet been defined. Recent evidence for molecular subtypes in PDAC with different sensitivity to EGFR inhibition supports this notion [10]. An additional noteworthy observation in our and the accompanying report was that the essential gatekeeper role of EGFR in PDAC development could be circumvented by concomitant inactivation of p53 but not the p16INK4a/p19ARF tumor suppressor [6, 7], perhaps invoking the stress response and genomic instability in the earliest stages of PDAC formation. Future investigations will need to focus on the precise signal profiles that dictate the use of EGFR inhibitors, tailored to the appropriate PDAC patient population and anticipating alternative modes of MEK/ERK activation likely to be associated with resistance. With all of these exciting new findings, the path is set for rethinking the role and regulation of oncogenic KRAS and EGFR-dependent signaling in PDAC for our ultimate goal to provide rational, basic research-driven and ultimately better therapies from our ever-increasing knowledge of the molecular secrets of this devastating disease.
